# Association of intravitreal and topical anti‐inflammatory therapies on short‐term anatomical and functional outcomes following epiretinal membrane surgery

**DOI:** 10.1111/aos.17430

**Published:** 2024-12-20

**Authors:** Pinja Sutinen, Idan Hecht, Minna Karesvuo, Sohee Jeon, Petteri Karesvuo, Raimo Tuuminen

**Affiliations:** ^1^ Helsinki Retina Research Group University of Helsinki Helsinki Finland; ^2^ Sackler School of Medicine Tel Aviv University Tel Aviv Israel; ^3^ Department of Ophthalmology Shamir Medical Center Tel Aviv Israel; ^4^ Department of Oral and Maxillofacial Diseases Kuopio University Hospital Kuopio Finland; ^5^ Vitreoretinal Service, Keye Eye Center Seoul South Korea; ^6^ Department of Ophthalmology Kuopio University Hospital Kuopio Finland; ^7^ Department of Ophthalmology Kymenlaakso Central Hospital Kotka Finland

**Keywords:** epiretinal membrane surgery, ERM peeling, NSAIDs, triamcinolone acetonide

## Abstract

**Purpose:**

Here we examine the association of anti‐inflammatory therapy with anatomical and functional outcomes of epiretinal membrane surgery.

**Methods:**

The study included consecutive patients having gone through epiretinal membrane surgery at Helsinki University Hospital, Finland, between 2017 and 2021. The association of perioperative intravitreal and postoperative topical anti‐inflammatory therapies with surgical outcomes was assessed.

**Results:**

In total, 214 eyes of 214 patients with a mean age of 71.2 ± 8.2 years were studied. At 1‐month, perioperative intravitreal use of triamcinolone acetonide (*n* = 27) was associated with a significant proportional and absolute improvement in foveal thickness (−15.9 ± 18.4% vs. −4.2 ± 25.6%, *p* = 0.003 and −86.2 ± 109.6 μm vs. −33.7 ± 94.3 μm, *p* = 0.004), and central subfield macular thickness (−14.7 ± 16.5% vs. −6.3 ± 16.9%, *p* = 0.009 and −80.6 ± 102.8 μm vs. −36.1 ± 75.9 μm, *p* = 0.004) when compared to those without triamcinolone acetonide. Furthermore, best‐corrected visual acuity (BCVA) gain showed a non‐significant yet corresponding trend favouring intraoperative intravitreal use of triamcinolone acetonide (0.21 ± 0.27 vs. 0.09 ± 0.28 LogMAR units, *p* = 0.062). Postoperative use of topical non‐steroidal anti‐inflammatory drugs (NSAIDs) (*n* = 36) as adjunct therapy showed no significant advantage in anatomical outcomes or BCVA gain when compared to those without NSAIDs (all *p* > 0.05). Triamcinolone acetonide remained significant for proportional and absolute improvement in foveal thickness (*p* = 0.019 and *p* = 0.006) and in central subfield macular thickness (*p* = 0.013 and *p* = 0.006) when controlled for confounding factors patient age and gender and topical NSAID use.

**Conclusions:**

Intraoperative intravitreal use of triamcinolone acetonide improved short‐term anatomical outcomes in patients who underwent epiretinal membrane surgery.

## 1 INTRODUCTION

The development of epiretinal membrane (ERM) is a common condition in the elderly with an incidence of around 10% in people over 70 (Aung et al., 2013; Fung et al., 2021). It is caused by metaplasia and proliferation of glial and retinal pigment epithelial cells alongside others, which leads to the formation of an abnormal membrane attached to the inner surface of the retina. ERM is often asymptomatic in its beginning stages but subsequently causes vision distortions including metamorphopsia and macropsia when the membrane wrinkles over time.

Surgery including pars plana vitrectomy (PPV) and peeling of the epiretinal membrane is the only treatment option available for correcting a vision impaired by ERM. The main complication of the operation include intraoperative haemorrhage, retinal tears, postoperative progressive nuclear sclerosis and macular oedema, which is strongly linked to postsurgical inflammation (Donati et al., 1998).

Numerous inflammatory mechanisms, including cellular inflammatory components and mediators such as cytokines and growth factors, are strongly associated with ERM formation (Joshi et al., 2013). The use of adjunct intravitreal steroids during ERM surgery is common, however, the current literature regarding the benefits of such use is inconclusive (Chen et al., 2022).

Triamcinolone acetonide is the most used intravitreal steroid in connection to ERM surgery, while dexamethasone which is typically used as a sustained‐release intravitreal implant (Ozurdex) is also widely used for managing macular oedema and inflammation (Chang et al., 2018; Hostovsky et al., 2020). Triamcinolone acetonide is a corticosteroid that is used both as a visualization agent and for its anti‐inflammatory effects caused by complex pathways and injected intravitreally (Sakamoto et al., 2002). It has been deemed as effective and safe as topical therapy (Paccola et al., 2007).

Production of prostaglandins in the eye has been noted as the main cause of inflammation following intraocular surgeries. Non‐steroidal anti‐inflammatory drugs (NSAIDs) reduce prostaglandin production by inhibiting cyclooxygenase‐1 and cyclooxygenase‐2 which are the key enzymes in prostaglandin biosynthesis (Ghlichloo & Gerriets, 2023) and thus widely used following various operations including ERM surgery.

Activation of inflammatory pathways can be associated with the formation of ERMs (Fung et al., 2021; Song et al., 2022; Yüksel et al., 2015). Following surgery for ERM, prolonged inflammation may predispose to a slower anatomical improvement postoperatively, and a higher likelihood of developing ERM recurrence (Fleissig et al., 2018; Ozturk et al., 2021).

In this study, we aimed to examine the possible influence of adjunct anti‐inflammatory therapy with perioperative intravitreal triamcinolone acetonide and postoperative non‐steroidal anti‐inflammatory drugs (NSAIDs) on ERM surgery outcomes.

## 2 METHODS

### 2.1 Study design

The study was approved by the Hospital District of Helsinki University Hospital, Helsinki, Finland (§42/2022; HUS/148/2022), and it adhered to the tenets of the Declaration of Helsinki. This study was a retrospective registry‐based cohort study of consecutive ERM surgeries performed between January 2017 and October 2021 at the Department of Ophthalmology, Helsinki University Hospital.

Prior to surgery and postoperatively, all patients underwent a complete ophthalmological examination including assessment of best‐corrected visual acuity (BCVA), anterior segment and fundus with slit‐lamp (biomicroscope), as well as retinal optical coherence tomography (OCT).

The main outcome was defined as a gain in BCVA at 1‐month. Anatomical changes were defined as secondary outcomes and included proportional and absolute changes in foveal, maximal and central subfield macular thickness (CSMT; here defined as mean thickness in the central 1000‐μm diameter area). All patients were followed for 1 month. According to the Helsinki University Hospital guidelines, patients routinely continued their long‐term follow‐up in the community‐based sector.

### 2.2 Clinical evaluation and postoperative care

Patients were diagnosed, operated and followed according to the Helsinki University Hospital guidelines. Clinical examination included visual acuity testing of both eyes at standardized room light conditions on decimal chart at 4 m. The diagnosis and preoperative assessment were performed by a vitreoretinal surgeon. OCT analysis was done in all cases pre‐ and postoperatively (Heidelberg Engineering GmbH, Heidelberg, Germany). International classification of diseases (ICD)‐coding was used to specify diagnosis; ICD‐10 H35.38# for macular preretinal fibrosis.

Postoperative care included routine use of levofloxacin 5 mg/mL eye drops (Oftaquix®, Santen Pharmaceutical Co. Ltd., Osaka, Japan) four times a day for 2 weeks and either combined dexamethasone phosphate 1 mg/mL and chloramphenicol 2 mg/mL eye drops (Oftan Dexa‐Chlora®, Santen Pharmaceutical Co. Ltd.) or prednisolone acetate (Pred Forte®, 10 mg/mL, AbbVie Inc., North Chicago, IL) four times a day for 1 month, depending on the surgeon's preference. Intraoperative intravitreal triamcinolone acetonide suspension (40 mg/mL, Triesence®, Novartis, Basel, Switzerland) at the end of surgery and postoperative nepafenac (3 mg/mL, Nevanac®, Novartis) once daily, diclofenac (1 mg/mL, Voltaren Ophtha®, Théa Laboratoires, Clermont Ferrand, France) t.i.d. or bromfenac (0.9 mg/mL, Yellox®, Bausch & Lomb, Laval, Canada) b.i.d. were given for one to 3 months to some of the patients depending on the surgeon's preference.

### 2.3 Inclusion and exclusion criteria

ERM surgeries with at least 1 month of follow‐up data were included in the study. Exclusion criteria were concomitant phacoemulsification surgery, prior vitrectomy for any reason, prior or present uveitis, macular hole, retinal vein occlusion, vitreomacular traction, proliferative diabetic retinopathy, anti‐VEGF treatment for any reason and missing data for the diabetic status.

### 2.4 Sample size calculation

For sample calculations, the primary outcome measure of BCVA gain between the groups was used. Important differences in BCVA are usually considered to range between 10 and 15 ETDRS letters (two to three ETDRS lines, equivalent to 0.2–0.3 LogMAR) (Danni et al., 2019; Fleissig et al., 2018). Here, two ETDRS lines (0.2 LogMAR) were considered as a clinically important difference. The standard deviation of visual gain was based on previous publications and set at 0.3 LogMAR. A sample size of 36 patients for each group (total of 72) was required to detect a significant difference between the groups with a significance level of 0.05 and a power of 80% using an independent model.

### 2.5 Statistics

To avoid biases arising from between‐eye correlation, a single eye of each patient was included in the study. Unless otherwise specified, data is presented as mean ± standard deviation (SD). BCVA decimal values were converted to logarithm of the minimum angle of resolution (logMAR) for statistical purposes. Clinical parameter distributions were tested for normality by the Shapiro–Wilk test. For categorical variables the *χ* ^2^ test was used, or the Fisher's exact test as appropriate. The independent *t*‐test was conducted for continuous variables with a normal distribution and the Mann–Whitney *U*‐test for variables with a non‐normal distribution. Analysis of Covariance was performed by the General Linear Model, in which the following factors were included: patient age and gender at the time of surgery, the use of intravitreal triamcinolone acetonide and the use of topical NSAIDs. *p*‐value for the effect of anti‐inflammatory medication on the existence of cysts (graded as follows: recovery from pre‐existing cyst −1; similar status between preoperative and postoperative timepoints 0; new‐onset cyst compared to preoperative status 1) was analysed by non‐parametric Mann–Whitney *U*‐test. Statistical analysis was performed using IBM SPSS Statistics 28 (IBM Corp. Armonk, NY). *p* < 0.05 on a two‐sided test was considered statistically significant.

## 3 RESULTS

The study included 214 eyes of 214 patients with a mean age of 71.2 ± 8.2 years. 51% of the patients were male and 49% female, with 9% overall currently smoking (*N* = 20). Baseline characteristics of the patients are available in Tables 1 and 2. Main baseline characteristics were comparable among those with and without perioperative triamcinolone acetonide (Table 1) and among those with and without postoperative NSAIDs (Table 2).

**TABLE 1:** Baseline characteristics regarding intravitreal triamcinolone acetonide.

Variable	Triamcinolone acetonide		*p*‐value
	No (*N* = 187)	Yes (*N* = 27)	
Age (years)	71.3 ± 8.3	71.2 ± 8.2	0.958
Diabetes	39 (21)	6 (22)	0.871
Current smoker	17 (9)	3 (11)	0.736
Gender (male: female)	98:89 (52:48)	12:15 (44:56)	0.439
Pseudophakia	90 (48)	13 (48)	0.998
Glaucoma	21 (11)	5 (19)	0.279
23:25:27G vitrectomy^a^	12:127:39 (6:68:21)	1:15:10 (4:56:37)	0.174
Topical NSAIDs (any)	30 (16)	6 (22)	0.422
*Clinical attributes*			
BCVA (LogMAR units)	0.42 ± 0.30	0.54 ± 0.42	0.153
Foveal thickness	452 ± 100	481 ± 85	0.150
CSMT	458 ± 82	484 ± 80	0.130
Max thickness	507 ± 105	533 ± 97	0.223

*Note*: Baseline characteristics among patients with and without intraoperative intravitreal triamcinolone acetonide at ERM surgery. Data is given as mean ± SD or absolute numbers (with proportions).

Abbreviations: BCVA, best‐corrected visual acuity; CSMT, central subfield macular thickness (mean thickness in the central 1000‐μm diameter area); ERM, epiretinal membrane; LogMAR, logarithm of the minimum angle of resolution; NSAIDs, non‐steroidal anti‐inflammatory drugs.

^a^ Data not available for 10 cases.

**TABLE 2:** Baseline characteristics regarding topical NSAIDs.

Variable	NSAIDs		*p*‐value
	No (*N* = 178)	Yes (*N* = 36)	
Age (years)	71.6 ± 8.1	69.4 ± 8.8	0.136
Diabetes	36 (20)	9 (25)	0.521
Current smoker	17 (10)	3 (8)	0.820
Gender (male: female)	91:87 (51:49)	19:17 (53:47)	0.856
Pseudophakia	86 (48)	17 (47)	0.905
Glaucoma	21 (12)	5 (14)	0.726
23:25:27G vitrectomy^a^	11:117:41 (6:66:23)	2:25:8 (6:69:22)	0.964
Intravitreal TA	21 (12)	6 (17)	0.422
*Clinical attributes*			
BCVA (LogMAR units)	0.43 ± 0.32	0.48 ± 0.33	0.498
Foveal thickness	457 ± 101	434 ± 87	0.211
CSMT	462 ± 85	447 ± 67	0.331
Max thickness	509 ± 102	505 ± 114	0.818

*Note*: Baseline characteristics among patients with and without intraoperative intravitreal triamcinolone acetonide at ERM surgery. Data is given as mean ± SD or absolute numbers (with proportions).

Abbreviations: BCVA, best‐corrected visual acuity; CSMT, central subfield macular thickness (mean thickness in the central 1000‐μm diameter area); ERM, epiretinal membrane; LogMAR, logarithm of the minimum angle of resolution; NSAIDs, non‐steroidal anti‐inflammatory drugs.

^a^ Data not available for 10 cases.

In patients treated with triamcinolone acetonide (*n* = 27) compared to those who were not, a significant proportional and absolute improvement in foveal thickness (−15.9 ± 18.4% vs. −4.2 ± 25.6%, *p* = 0.003, Table 3 and −86.2 ± 109.6 μm vs. −33.7 ± 94.3 μm, *p* = 0.004, Table S1), CSMT (−14.7 ± 16.5% vs. −6.3 ± 16.9%, *p* = 0.009, Table 3 and −80.6 ± 102.8 μm vs. −36.1 ± 75.9 μm, *p* = 0.004, Table S1) and maximal thickness (−11.6 ± 18.7% vs. −4.9 ± 17.1%, *p* = 0.043, Table 3 and −74.1 ± 122.7 μm vs. −34.7 ± 98.1 μm, *p* = 0.030, Table S1) was observed.

**TABLE 3:** Adjunct perioperative intravitreal triamcinolone acetonide.

Triamcinolone acetonide	− (*N* = 187)	+ (N = 27)	*p*‐value
BCVA gain (LogMAR)	0.09 ± 0.28	0.21 ± 0.27	0.062
Change (% from baseline)			
Foveal thickness	‐4.2 ± 25.6	−15.9 ± 18.4	0.003
CSMT	−6.3 ± 16.9	−14.7 ± 16.5	0.009
Maximal thickness	−4.9 ± 17.1	−11.6 ± 18.7	0.043

*Note*: Functional and anatomical 1‐month outcomes among patients undergoing ERM surgery. Data is given as mean ± SD.

Abbreviations: BCVA, best‐corrected visual acuity; CSMT, central subfield macular thickness (mean thickness in the central 1000‐μm diameter area); LogMAR, Logarithm of the Minimum Angle of Resolution.

BCVA gain showed a non‐significant yet corresponding trend for further improvement among patients treated with triamcinolone acetonide (0.21 ± 0.27 LogMAR units) compared to those who were not (0.09 ± 0.28 LogMAR units, *p* = 0.062). In a multivariate analysis for BCVA gain adjusted for the presence of diabetes and pseudophakic status, patients treated with triamcinolone acetonide showed consistent results with a non‐significant similar trend for BCVA improvement (OR for BCVA gain above the median: 2.312, 95% CI: 0.929–5.755, *p* = 0.072).

Treatment with postoperative topical NSAIDs including nepafenac (*n* = 29), diclofenac (*n* = 2), bromfenac (*n* = 2) or any NSAID (*n* = 33) showed no significant advantage in any anatomical outcome tested or in BCVA gain (Table 4, Table S1).

**TABLE 4:** Adjunct postoperative topical NSAIDs.

Nepafenac	− (*N* = 185)	+ (N = 29)	*p*‐value
BCVA gain (LogMAR)	0.12 ± 0.27	0.03 ± 0.32	0.144
Change (% from baseline)			
Foveal thickness	−6.5 ± 24.9	−0.7 ± 26.3	0.142
CSMT	−7.6 ± 17.1	−5.4 ± 17.1	0.258
Maximal thickness	−6.0 ± 16.9	−3.5 ± 20.9	0.270

Any NSAID^a^	− (N = 178)	+ (N = 36)	*p*‐value
BCVA gain (LogMAR)	0.11 ± 0.26	0.09 ± 0.36	0.760
Change (% from baseline)			
Foveal thickness	−6.7 ± 25.2	−0.6 ± 24.3	0.091
CSMT	−8.1 ± 17.2	−3.7 ± 16.1	0.077
Maximal thickness	−6.3 ± 17.0	−2.6 ± 19.4	0.145

*Note*: Functional and anatomical 1‐month outcomes among patients undergoing ERM surgery. Data is given as mean ± SD.

Abbreviations: BCVA, best‐corrected visual acuity; CSMT, central subfield macular thickness (mean thickness in the central 1000‐μm diameter area); LogMAR, Logarithm of the Minimum Angle of Resolution.

^a^ Includes patients treated with nepafenac, diclofenac or bromfenac.

Analysis of covariance revealed that the effect of triamcinolone acetonide remained significant for proportional and absolute improvement in foveal thickness (*p* = 0.019 and *p* = 0.006, respectively, Table 5) and in CSMT (*p* = 0.013 and *p* = 0.006, respectively, Table 5) when controlled for confounding factors patient age and gender and topical NSAID use.

**TABLE 5:** Analysis of covariance.

	*p*‐value
*BCVA gain (LogMAR)*	
Age	0.094
Gender	0.402
Triamcinolone acetonide	0.077
Topical NSAID	0.599

Change	(% from baseline)	(μm from baseline)
*Foveal thickness*		
Age	0.487	0.226
Gender	0.235	0.170
Triamcinolone acetonide	0.019	0.006
Topical NSAID	0.191	0.099
*CSMT*		
Age	0.404	0.225
Gender	0.113	0.110
Triamcinolone acetonide	0.013	0.006
Topical NSAID	0.165	0.113
*Maximal thickness*		
Age	0.082	0.034
Gender	0.145	0.324
Triamcinolone acetonide	0.055	0.056
Topical NSAID	0.295	0.474

*Note*: Functional and anatomical 1‐month outcomes among patients undergoing ERM surgery by the analysis of covariance (ANCOVA) to control confounding factors. Gain in LogMAR units were treated as continuous variables in General Linear Model.

Abbreviations: BCVA, best‐corrected visual acuity; CSMT, central subfield macular thickness (mean thickness in the central 1000‐μm diameter area); LogMAR, Logarithm of the Minimum Angle of Resolution.

Finally, we examined the association of triamcinolone acetonide and topical NSAIDs administration on the existence of foveal cysts (cystoid macular oedema). Perioperative intravitreal triamcinolone acetonide was favourable against the incidence of cysts (*p* = 0.011, Table S2), whereas topical NSAIDs were not (*p* = 0.418, Table S2).

## 4 DISCUSSION

Among patients who went through ERM surgery, intraoperative intravitreal use of triamcinolone acetonide was associated with better anatomical outcomes, whereas topical NSAID anti‐inflammatory adjunct therapy did not.

ERM surgery is widely considered to improve outcomes in extreme cases of ERM (Güngel et al., 2015; Yusuf et al., 2021). In general, greater foveal thickness values may indicate localized traction and more severe ganglion cell damage (Cobos et al., 2013). The presence of concurrent macular oedema has been shown to be a poor prognostic factor for macular thickness improvement (Grzybowski, Kanclerz, et al., 2019). Inflammation is a key mediator of pathogenetic processes responsible for adverse outcomes postoperatively in a wide range of ocular surgeries (Kuppermann et al., 2014). Two types of intravitreal steroids are administered in daily practice: triamcinolone and dexamethasone, while some other commercially available formulations are not candidates for intravitreal administration due to their toxicity (Kuppermann et al., 2014). Dexamethasone is a more potent steroid than triamcinolone in terms of anti‐inflammatory action, and inhibition of vascular permeability factors and development of macular oedema (Grzybowski, Brockmann, et al., 2019; Grzybowski & Kanclerz, 2019). However, the effective duration of the two corticosteroids differ, as dexamethasone is delivered as a slow‐release dexamethasone implant (SRDI; Ozurdex®, Allergan Inc., Irvine, CA), while triamcinolone acetonide is administered as an intraocular suspension or subconjunctival depot (Lindholm et al., 2019). A single intravitreal dexamethasone implant (0.7 mg) can maintain high vitreous concentrations for up to 60 days and low concentrations up to 6 months after administration (Chang‐Lin et al., 2011). In contrast, the mean elimination half‐life of intravitreal or subconjunctival triamcinolone is much shorter, and in the case of intravitreal administration in vitrectomized eyes, even more so (Beer et al., 2003). Still, the effect of intravitreal steroids might be particularly important in the early postoperative period; moreover, eyes undergoing vitrectomy for ERM might undergo a limited but not complete vitrectomy.

The current literature regarding the benefits of applying adjunct intravitreal steroids in ERM surgery is inconclusive (Table 6). A single comparative study has reported greater visual gain after the use of SRDI in advanced idiopathic ERMs. Two other studies reported a greater reduction of macular thickness following an intravitreal injection of triamcinolone acetonide 1 mg or 4 mg. Other investigations demonstrated a transient benefit in terms of reduction of macular thickness or no benefit at all (Ahn et al., 2012; Angermann et al., 2018; Guidi et al., 2018; Iovino et al., 2019; Lai et al., 2011; Narayanan et al., 2020; Savastano et al., 2020; Sella et al., 2015). Despite this the use of adjunct intravitreal steroids during ERM surgeries is common, creating a need for more research on the subject. The findings of this study support the intraoperative use of triamcinolone acetonide due to lowered macular thickness after administration. Complementary results have been recorded before, where the use of triamcinolone acetonide in ERM surgery similarly resulted in a significant decrease in central macular thickness despite not considerably influencing visual acuity (Angermann et al., 2018). Yonekawa et al. (2016) reported even greater results with triamcinolone acetonide improving visual and anatomical outcomes significantly when injected intravitreally (Yonekawa et al., 2016), while Leisser et al. (2022) stated that periocularly applied triamcinolone acetonide showed prevention of early transient macular oedema (Leisser et al., 2022).

**TABLE 6:** Comparative studies reporting outcomes of vitrectomy with epiretinal membrane peeling and adjunctive intravitreal steroid administration.

Study	Design	Surgical technique	Steroid	No. of eyes (patients)	Outcome
Savastano et al. (2020)	Retrospective analysis: vitrectomy for idiopathic ERM removal with or without SRDI	25G vitrectomy with ERM and ILM peeling	SRDI	20 vs. 20 (20 vs. 20)	SRDI accelerated improvement in visual acuity 15 days after surgery, bo no evidence on anatomical or functional improvement was noted 30 and 90 days after surgery
Narayanan et al. (2020)	Retrospective analysis: vitrectomy for idiopathic ERM removal with or without SRDI	25G vitrectomy with ERM without and ILM peeling	SRDI	15 vs. 15 (15 vs. 15)	Better VA and lower retinal thickness at 1 month in patients receiving SRDI. However, no significant differences at 6‐month follow‐up
Iovino et al. (2019)	Retrospective analysis: vitrectomy for advanced (stage 3–4) ERM removal with or without SRDI	25G vitrectomy with ERM and ILM peeling	SRDI	20 vs. 20 (20 vs. 20)	At 3 and 6 months the visual gain and decrease macular thickness was better in SRDI group
Angermann et al. (2018)	Retrospective analysis: vitrectomy for idiopathic ERM removal with or without IVTA	23 or 25G vitrectomy with ERM and ILM peeling	IVTA 1 mg	20 vs. 21 (20 vs. 21)	IVTA did not influence the visual outcome at 6 months, however, lower foveal thickness was observed after IVTA
Guidi et al. (2018)	Retrospective analysis: vitrectomy for idiopathic ERM removal with or without SRDI	25G vitrectomy with ERM and ILM peeling	SRDI	30 vs. 30 (30 vs. 30)	No significant difference in visual acuity, macular thickness or volume at 6‐month follow‐up
Sella et al. (2015)	Retrospective analysis: vitrectomy for idiopathic ERM removal with or without IVTA	23 or 25G vitrectomy with ERM and with or without ILM peeling	IVTA 4 mg	26 vs. 49 (26 vs. 49)	Macular thickness was further reduced in patients receiving IVTA, but with no difference in visual acuity 3 months after surgery
Ahn et al. (2012)	Retrospective analysis: vitrectomy for idiopathic ERM removal with or without IVTA	23 or 25G vitrectomy with ERM and with or without ILM peeling	IVTA 4 mg	27 vs. 31 (27 vs. 31)	IVTA did not affect foveal thickness and functional recovery for 3 months after surgery
Lai et al. (2011)	Retrospective analysis: vitrectomy for idiopathic ERM removal with or without IVTA	Vitrectomy with ERM and without ILM peeling	IVTA 2 mg	71 vs. 27 (71 vs. 27)	IVTA produced no benefits in terms of improvement in macular thickness or visual acuity, but did increase the risk of increased IOP 12 months after surgery

*Note*: Studies reporting on outcomes of epiretinal membrane surgery and adjunctive intravitreal steroid administration. Case reports or case series were not included.

Abbreviations: ERM, epiretinal membrane; ILM, internal limiting membrane; IOP, intraocular pressure; IVTA, intravitreal triamcinolone acetonide; SRDI, slow‐release dexamethasone implant (Ozurdex®, Allergan Inc., Irvine, CA).

However, the study found no further evidence to support the use of postoperative topical NSAIDs following surgery for ERM. Although it seems a reasonable conclusion that the mechanisms of NSAIDs would be beneficial following the surgery, previous research on the subject has shown similar results concluding that the use of topical NSAIDs did not improve surgical outcomes including macular thickness among other factors (Kim et al., 2024; Mandelcorn et al., 2022). A study by Schoenberger et al. (2011) reported against this that the use of topical nepafenac reduced macular volume more rapidly following ERM surgery (Schoenberger et al., 2011). A larger study on NSAIDs in connection to ERM surgery is required to conclude whether the use is beneficial for cost‐effectiveness and the potential, although usually relatively minor, side effects (Ghlichloo & Gerriets, 2023).

This study has some limitations. As a tertiary unit, our patients are referred from numerous centres across Southern Finland, including Central Hospitals and private practitioners. The follow‐up time in the study was just 1 month since the patients continued their check‐ups in the community‐based sector along the guidelines of Helsinki University Hospital. Due to these circumstances, long‐term data was not available. Nevertheless, 1‐month outcomes after ERM peeling well resonates with the long‐term prognosis. Improvement in retinal thickness and visual acuity can be observed up to 1‐year postsurgery (Hecht et al., 2017; Rii et al., 2014), but it has been shown that early anatomical changes following the operation predict long‐term anatomical outcomes well (Vingopoulos et al., 2021). While baseline variables were mostly similar between the groups, there remains residual confounding that cannot be controlled for. Finally, the amount of perioperative intravitreal triamcinolone acetonide injection was not standardized and may have varied according to the surgeon's preference. Despite the limitations which are characteristic of retrospective study design, we believe this work can provide important insights into the pertinent clinical question regarding anti‐inflammatory adjunct therapy.

In conclusion, in a patient cohort that underwent ERM surgery, perioperative intravitreal use of triamcinolone acetonide was associated with improved short‐term anatomical outcomes in the form of decreased foveal and central subfield macular thickness.

## FUNDING INFORMATION

The authors have neither proprietary nor commercial interests in any medications or materials discussed in this study. Dr. Tuuminen is a scientific adviser (advisory board, honoraria) to Alcon Laboratories, Inc., Allergan, Inc., Bayer AG, F. Hoffmann‐La Roche Ltd. and Novartis AG and has received clinical trial support (study medicines) from Bayer AG and Laboratoires Théa.

## CONFLICT OF INTEREST STATEMENT

No conflicting relationships exist for any author.

## Supporting information


Table S1.

Table S2.
