# Supplementary material for: Association of intravitreal and topical anti‐inflammatory therapies on short‐term anatomical and functional outcomes following epiretinal membrane surgery
Source: Acta Ophthalmol. 2024 Dec 20;103(4):416–22. doi: 10.1111/aos.17430 (PMC12069963; doi:10.1111/aos.17430)
Supplement: Supplementary file 1 — Table S1. [file AOS-103-416-s001.docx]

**Supplement Table 1.** Adjunct anti-inflammatory medication.

| **Triamcinolone acetonide** | -  (N=187) | +  (N=27) | *P*-value |
| --- | --- | --- | --- |
| Change (µm from baseline) |  |  |  |
| foveal thickness | -33.7±94.3 | -86.2±109.6 | .004 |
| CSMT | -36.1±75.9 | -80.6±102.8 | .004 |
| Maximal thickness | -34.7±98.1 | -74.1±122.7 | .030 |

| **Nepafenac** | -  (N=185) | +  (N=29) |  | *P*-value |
| --- | --- | --- | --- | --- |
| Change (µm from baseline) |  |  |  |  |
| foveal thickness | -43.7±97.4 | -18.6±97.8 |  | .205 |
| CSMT | -43.4±81.1 | -30.3±79.7 |  | .427 |
| Maximal thickness | -40.7±95.4 | -33.0±140 |  | .711 |
|  |  |  |  |  |
| **Any NSAID*** | -  (N=178) | +  (N=36) |  | *P*-value |
| Change (µm from baseline) |  |  |  |  |
| foveal thickness | -45.4±98.2 | -15.1±91.8 |  | .093 |
| CSMT | -45.6±81.6 | -21.8±74.9 |  | .112 |
| Maximal thickness | -42.4±96.2 | -25.9±128 |  | .384 |

Effects of adjunct anti-inflammatory medication on anatomical 1-month outcomes among patients undergoing ERM surgery. Absolute change (µm) from baseline is given as mean ± SD. CSMT; central subfield macular thickness (mean thickness in the central 1000-$\mu$m diameter area). *Includes patients treated with nepafenac, diclofenac or bromfenac.
