# Supplementary material for: Association of intravitreal and topical anti‐inflammatory therapies on short‐term anatomical and functional outcomes following epiretinal membrane surgery
Source: Acta Ophthalmol. 2024 Dec 20;103(4):416–22. doi: 10.1111/aos.17430 (PMC12069963; doi:10.1111/aos.17430)
Supplement: Supplementary file 2 — Table S2. [file AOS-103-416-s002.docx]

**Supplement Table 2.** Pre and postoperative cysts (cystoid macular edema) in regard anti-inflammatory treatment

| **Perioperative intravitreal**  **triamcinolone acetonide** | -  (N=187) | +  (N=27) |  | *P*-value |
| --- | --- | --- | --- | --- |
| Incidence (%) |  |  |  |  |
| Preoperative cysts | 29 | 69 |  |  |
| Postoperative cysts | 38 | 52 |  |  |
| Overall effect |  |  |  | .011 |
|  |  |  |  |  |
| **Any postoperative**  **topical NSAID** | -  (N=178) | +  (N=36) |  | *P*-value |
| Incidence (%) |  |  |  |  |
| Preoperative cysts | 29 | 58 |  |  |
| Postoperative cysts | 34 | 68 |  |  |
| Overall effect |  |  |  | .418 |

Incidence of cysts on spectral-domain optical coherence tomography (SD-OCT) before ERM peeling and at 1-month. P-value for the effect of anti-inflammatory medication on the existence of cysts (graded as follows: recovery from pre-existing cyst -1; similar status between preoperative and postoperative timepoints 0; new-onset cyst compared to preoperative status 1) was analyzed by non-parametric Mann-Whitney U test.
